# Parents’ Adherence to Childhood Screening Tests and Referrals: A Retrospective Cohort Study with Randomized Sampling

**DOI:** 10.3390/ijerph19106143

**Published:** 2022-05-18

**Authors:** Anat Amit Aharon

**Affiliations:** Department of Nursing, School of Health Professions, Tel Aviv University, Tel Aviv 6139001, Israel; anatamit@tauex.tau.ac.il

**Keywords:** parent’s adherence, physician/nurse referral, screening test, well-child visit

## Abstract

Routine timely examinations of well-child health are important for achieving children’s good health outcomes. Nevertheless, there is evidence of low compliance with well-child visit recommendations. The aim of the study was to examine potential factors associated with parents’ nonadherence to routine childhood screening tests and their acting on further referrals following unusual findings. A retrospective cohort study was conducted among 14,348 children born in 2016–2017 and registered at mother–child health clinics in a large city in Israel. A sample of 844 children was randomly selected. Screening tests at the age of two months and nine months were examined. A multiple logistic regression examined potential factors associated with nonadherence to screening tests and to further referral for evaluation. Lower adherence to screening tests was found among parents of nine-month-old children, but adherence was higher for nurses’ screening tests than for those of physicians. Children born in a complex delivery process, older mothers with a higher number of children, and Israeli citizens were at risk of not undergoing screening tests. Fewer children in the family and initial physician’s findings were the only explanation for acting to referrals. In order to promote children’s health outcomes and public health, health policymakers should conduct campaigns to convince parents of the importance of screening tests and of adherence to referrals with the aim of ensuring their children’s wellbeing throughout the life cycle.

## 1. Introduction

Routine timely examinations of well-child health by pediatricians or nurses are an important tool for achieving children’s good health outcomes [1,2] and wellbeing [3]. The American Academy of Pediatrics (AAP) recommends 17 visits to a pediatrician until age 6 for healthy children [4]. In these visits, the child is examined for primary and secondary preventive care, including vaccinations, physical examinations, growth parameters, and monitoring developmental milestones. Compliance with routine visits in early childhood may reduce the need for hospitalization, prevent childhood diseases and injuries, improve a child’s psychomotor skills, as well as improve parenting skills [2]. Diagnosis and therapeutic intervention in early childhood are most effective for improving children‘s health outcomes and quality of life at maturity [5,6,7]. Nevertheless, there is evidence of low compliance with well-child visit recommendations [8], low physician referral for further diagnosis [9], and low parent adherence to referrals, with a wide range of influencing factors, such as low socioeconomic status, lack of health insurance, and lack of awareness [10,11]. Wolf et al. (2018) [12] found that Hispanic and Asian American children attended fewer routine well-child visits compared to non-Hispanic white children (OR = 1.38, CI = 1.36–1.40; OR = 1.36, CI = 1.32–1.40, respectively). A low level of parent knowledge regarding cognitive and language development in early childhood was associated with low adherence to nine-month visits to well-baby clinics [13]. In Europe, Moller et al. (2016) [14] reported that foreign children have a lower intake of vaccination and routine child health examinations compared to native-born children. A study conducted in Israel in 2004 found that 9% of Jewish children and 8% of children of Arab ethnicity did not adhere to physician examinations, but all the children adhered to nurse examinations. Children of mothers with a higher education level and with a higher number of children in the family, both Jewish and Arab, adhered less to MCHC visits compared to those with mothers with a lower education and fewer children in the family. Moreover, children of Arab ethnicity adhered less to hearing screening tests compared to Jewish children (75% vs. 86%) [15]. Notably, since 2004, routine visit and screening test protocols at MCHCs have dramatically changed. A study from 2018 found no differences between Israeli children and refugee children regarding the number of visits to MCHCs and vaccination rates. Nevertheless, Israeli children had a higher rate of performing blood screening tests for hemoglobin at one year [16]. As far as known, there are no other new studies regarding adherence to visits to MCHCs in Israel.

### Preventative Health Services in Israel

In Israel, preventive health services operate according to the Ministry of Health policy through mother–child health clinics (MCHCs) with nationwide services. The preventive health services are provided under the National Health Insurance Law, 1995 [17]. According to the current policy, a child should visit a MCHC 6–7 times during the first year of life, twice in the second year of life, and then once a year until age 6. All visits include observation by a public health nurse. Nurses are in charge of the vaccination program, anthropometric measurements, development milestone screening tests (ST), nutrition guidance, anticipatory parental training, and health promotion programs [18]. Children should be examined by a pediatrician at ages of two, nine, and 18 months, and 5 years. Pediatricians concentrate on comprehensive physical STs, “head-to-toe examinations” [19]. Public health nurses focus on developmental and growth screening tests with formal validated screening tools. For example, qualified nurses use the “Tipat Halav Israeli Screening” (THIS) developmental scale. This age-based scale examines 59 developmental milestones in four developmental domains (fine and gross motor skills, language, and social skills). Achievement rate by child’s age is calculated for each milestone. According to the achievement rate the nurse is able to make a decision on whether to refer the child for further diagnostic tests [20]. The MCHC focuses only on preventive health according to the Ministry of Health protocol (#3/2004). When a suspicious physical or developmental finding is discovered at the MCHC, the child is referred for further diagnosis and treatment at the health plans (HMO) where he or she is insured.

Preventive health services in Israel are provided free-of-charge, are universal, and include noncitizens, e.g., children of asylum seekers [18]. Nevertheless, acute and curative health services in Israel are provided by the four HMOs under payment of a monthly health tax. Under the National Health Insurance Law, all citizens have health insurance. Children of foreign workers and asylum seekers are not citizens, and hence must purchase private or public health insurance. About 35–40% of foreign children have health insurance [21].

The proportion of children (aged 0–6) registered at MCHCs is estimated at 95% of all children born in Israel [22]. Stein-Zamir, Shoob, and Zimmerman (2017) [23] reported that 89.5% of children registered at MCHCs saw a physician at least once. Parents who did not see the MCHC physician were younger and had fewer children in the family. As far as known, there are no other formal data or studies that examined parental adherence to screening tests at two months and nine months of age in MCHCs.

Most of the literature focuses on developmental disorders as the main reason for referring a child for further diagnosis [24,25,26]. Few studies have focused on physical problems or growth disorders as the cause for referral [27]. In addition, there is scant information regarding parental adherence to routine screening tests at two months and nine months of age at MCHCs and the potential factors associated with parent nonadherence and on the other hand, there is a lack of information on the health outcomes of screening tests performed at MCHCs. In order to bridge this gap, the objectives of this study were:

(a) To explore the potential factors associated with parents’ nonadherence with routine screening tests performed by physicians and nurses at MCHCs.
(b) To explore parents’ response to referrals for further diagnostic evaluation and health outcomes of diagnostic tests.

## 2. Materials and Methods

### 2.1. Data Source

A retrospective study conducted among a cohort of children born in 2016–2017 in the second largest city in Israel. The data were collected from the computerized medical files of children registered at MCHCs, with no identifying details. A cohort of 2017 children was chosen for examination at age 2 months and a cohort of 2016 children for examination at age 9 months. Overall, 14,348 children born in 2016–2017 were registered at MCHCs. The representative sampling was calculated by assuming a 5% margin of error, power of 80%, two-tailed, *p* value < 0.05 of the test and a confidence interval (CI) of 95%. Assuming child mobility and a low rate of referrals, it was decided to increase the sample size by 15%. The sample was randomly selected by computer software from the list of children registered at MCHCs. The final sample included 844 children (397 born in 2016 and 447 in 2017). Data collection was conducted from January to August 2019.

### 2.2. Measures of Variables

Three groups of variables were collected:

1. Child information, including gender, number of children in the pregnancy, weeks of pregnancy (weeks and days), delivery process (vaginal, caesarian section, or complex).
2. Mother’s information, including mother’s age, mother’s citizenship (Israeli citizen/foreign worker or asylum seeker), education level (academic/not academic), religion (Jewish/Arab), marital status (married/not married), number of children in the family.
3. Physician and nurse examination, including date of two-month and nine-month ST, outcome of ST (no finding/suspicion of unusual finding), whether the outcome was the first observation (yes, it is the initial finding/no, the child was diagnosed previously. This measure was relevant only for physicians), referral for further diagnosis (yes/no), parents acted on the referral (yes/no), outcome of further diagnosis (no finding/pathological finding).

Physicians’ screening tests include physical examination at two months and nine months of age. In these screening tests the physician systematically examines all the body systems. For example, the physician scans the skin system to identify suspicious lesions, discoloration, or skin tag. The results are summarized for each body system: “No suspicious physical finding or suspicion of unusual finding (describing the finding)”. At his discretion, the physician decides whether the child should be referred for further diagnosis. The test at the age of two months and nine months is the same.

Nurse screening tests include the growth and developmental domain according to the child’s age. Growth screening includes anthropometric measurements. The developmental screening test includes the THIS milestone, according to the child’s age [20]. The results are summarized for “no finding/suspicion of unusual finding (describing the finding)”. At her discretion, the nurse decides whether the child should be referred for further diagnosis.

### 2.3. Data Analyses

Descriptive statistics were used to characterize the research sample. An χ^2^ test was performed to examine associations between compliance with examination by a physician or nurse and the sociodemographic variables and Student’s *t*-test for examining differences between children who did and did not undergo the ST. Finally, a logistic regression was performed to analyze the potential factors associated with not complying with the ST protocol. Values of *p* < 0.05 were considered significant. All analyses were performed using SPSS version 25 (IBM, Armonk, NY, USA).

## 3. Results

The mean birth age of the children was 39.2 weeks. Most of the pregnancies were single (94.9%) and one was triple. Sixty-eight percent of the deliveries were vaginal and the rest were caesarian deliveries or complex deliveries, with some instrumental deliveries. Most of the children were born with no medical problems (69.6%) but 30.4% were born with mild-to-severe pathological findings, for example premature, torticollis, tongue-tied, systolic murmur, undescended testicles, respiratory distress, hemangiomas, skin tag, hydrocephalus, and others. Few children were born with multiple problems (Appendix A).

Children aged two months (2017 cohort) underwent more STs performed by physicians and nurses than children aged nine months (2016 cohort) to a statistically significant degree (81.9% vs. 72.8%, *p* = 0.002; 97.8% vs. 87.7%, *p* = 0.001, respectively, Table 1).

### 3.1. Screening Test Outcomes

Table 2 describes the ST outcomes (physicians and nurses). Most of the children underwent physician and nurse STs, but children underwent more nurse STs than physician STs (93.0% vs. 77.6%, *p* = 0.0001), nurses referred more children than did physicians (86.8% vs. 35.9%, *p* = 0.0001), and the parents acted on nurses’ referrals more than on physicians’ (88.6% vs. 64.9%, *p* = 0.0001), to a statistically significant degree.

Analysis of the differences between children who did or did not undergo STs performed by physicians revealed an association between *not* undergoing a physician’s ST and single pregnancy, children born in a vaginal delivery, children of Israeli citizen mothers, older mothers, academics, and Jewish mothers, and a higher number of children in the family (Table 3). With regard to nurses, an association was found between not undergoing a nurse ST and mother’s higher age, being an Israeli citizen, and being Jewish (Table 3).

A multiple logistic regression was conducted to explore the association between the research variables and the likelihood of compliance with STs performed by physicians/nurses. Variables that were significant in previous statistical tests were included in the multiple logistic regression. Therefore, the explanatory variables for physician’s ST were number of children in the pregnancy, the delivery process, mother’s age, number of children in the family, mother’s origin, mother’s educational level, and religion. The explanatory variables for nurse’s ST were mother’s age, mother’s origin, and religion.

The multiple logistic regression revealed that children born in a complex delivery were 1.70 times more likely to avoid adhering to a physician’s ST than children born in a vaginal delivery (CI: 1.14–2.54). Children of older mothers were 0.96 times more likely to not comply with a physician’s ST than children of younger mothers (CI: 0.27–0.99). In addition, children from families with a higher number of children and Israeli citizen children were more likely to avoid adhering to a physician’s ST than children from families with fewer children and foreign or asylum seekers’ children (OR= 0.84, CI: 0.24 − 0.97; OR = 3.95, CI: 1.73–9.04, respectively). Children of older mothers and Israeli citizen children showed less adherence to nurses’ ST than children of younger and foreign or asylum-seeker mothers (OR = 0.93, CI: 0.88–0.98; OR = 10.33, CI: 1.17–19.61, respectively) (Table 4).

### 3.2. Parents’ Response to Referrals

Physicians referred 94 children for further medical diagnosis, but only 61 (64.9%) acted on the referrals. Statistically significant differences were found between the groups only in the number of children in the family and whether the outcome of the ST was an initial finding by the physician. Parents with fewer children in the family acted on physician referrals more than parents with more children in the family (M = 1.62 vs. M = 2.06, F = 0.57, *p* < 0.01). Furthermore, 70.7% of parents of children with initial findings acted on the referrals, compared to 42.1% of parents of children with no initial finding (*p* < 0.01). No other statistically significant differences were found between the groups (Table 5).

Nurses referred 79 children for further medical diagnosis, but only the parents of 70 children (88.6%) acted on the referrals. There was no statistically significant difference between the group who acted and who did not act on nurses’ referral (Table 5).

## 4. Discussion

The current study explored the differences between children whose parents adhered to routine STs by physicians and public health nurses and acted on their referrals and children whose parents did not comply. The findings indicated a high rate of compliance with physicians’ and nurses’ ST, but the parents of two-month-old children adhered to the recommendations more than those of nine-month-old children, both for physicians’ and for nurses’ ST. Previous studies found that children under one year old show higher compliance with recommendations for well-child visits compared to older children [2,8]. The current study elaborated on these findings, showing that parents of nine-month-old infants are significantly less compliant with physicians’/nurses’ ST than parents of two-month-old infants. Nevertheless, there is a higher rate of compliance by parents of nine-month-old infants with nurses’ STs than with physicians’ STs. This may suggest that parents perceived STs at age nine months as less important or less relevant for their child, particularly with regard to physicians’ observations. In addition, it may indicate that parents are less aware of the importance of STs at age nine months, such as early detection of abnormal physical or developmental health conditions, and that early intervention may ensure a better prognosis than will a delay in treatment [28]. This finding may suggest that it is necessary to raise parents’ awareness of the importance of performing screening tests, particularly at nine months of age and older. Darling et al. (2020) [29] argue that the first 1000 days (until age two) are a critical window of opportunities for monitoring and early intervention to improve child development for long-term good health for the child and community.

The greater adherence to nurses’ observations than to physicians’ is associated with the nature of preventive health services in Israel. MCHCs are well-established in Israel and are all managed by public health nurses with excellent professional training [18,22]. MCHC health services are identified by parents as a nurse service and are less identified with physicians. Nurses are highly valued and trusted by the public [15]. On the other hand parents, and particularly younger parents, and less aware of the physician’s role [23], as he or she comes to the MCHC only for a few hours a week, and therefore the parents make less use of physicians’ STs.

In the current study, the physicians and nurses chose not to send all the children who did not pass the ST for further diagnosis. Only one-third (35.9%) of the children with suspicious abnormalities received a physician’s referral. This finding is consistent with previous studies suggesting that many children with a suspicious developmental abnormality are not referred for further diagnosis [30] due to several barriers, including lack of awareness of continued treatment in the community [2]. Other studies reported a physician referral rate of 14% to 61% [9,31]. In the current study, physician referrals were for medical problems such as skin or cardiac abnormalities but not for developmental abnormalities. Furthermore, MCHC physicians are well aware of the option of continued treatment in the community under the National Health Insurance Law, although in a recent study in Israel, some pediatricians reported a lack of knowledge regarding developmental ST practices [26]. Furthermore, it can be assumed that some of the physicians’ findings were minor abnormalities with no need for further intervention. Some of the unreferred children had already been diagnosed with a pathological condition and were receiving treatment. On the other hand, MCHC nurses gave referrals for anthropometric measurements and developmental abnormalities. Nurses referred most of the children (86.8%) for further diagnosis. More importantly, for more than half the children who had suspicious physical abnormalities, the physician at the MCHC was the first to raise the suspicion, and therefore they are called initial findings. “Initial finding” was one of the two variables associating between a physician’s referral and parents’ acting on the referral. Dinkevich and Ozuah (2002) [27] estimated that the rate of initial physical findings uncovered in routine visits by a physician is only 2–3% of children aged 0–18 years. To the best of knowledge, this variable has yet to be thoroughly examined. Therefore, the finding in the current study emphasizes the importance outcome of physicians’ examinations at the MCHC.

Another aspect is related to the parents. In the current study, not all the parents complied with physician or nurse STs and not all the parents who received a referral to continue the diagnosis process acted on the referral (but a higher response was evident for nurse referrals). Stein-Zamir et al. (2017) [23] found that 3.8% of parents reported that visits to physicians at the MCHC are unnecessary. Other barriers to parents’ low compliance with physician or nurse STs included communication barriers [30], low parent socioeconomic status [2], minority or ethnic barriers [32], and lack of health insurance [1,11]. These barriers could deepen health disparities and health inequity [2,32]. In the current study, the barrier of lack of health insurance seems less relevant for several reasons:

(a) The national health insurance law applies to all children in Israel who are citizens, including minority citizens, with the exception of foreign workers’ children. This means that all citizen children necessarily receive health care.
(b) Children of foreign workers/asylum seekers, of whom only some had health insurance, were significantly more likely to undergo physician and nurse examinations than Israeli citizen children.
(c) No differences were found between children whose parents did or did not comply with physician/nurse referrals by their health insurance (citizen children compared to foreign children).

Moreover, the current study indicated that a complex delivery process, being Israeli citizens, as well as higher mother’s age and having more children in the family, are potential factors associated with not complying with the ST at the MCHC. The findings in the literature are inconsistent: some studies found that younger mothers were less compliant with well-child visits [11,23] and others found that older mothers were less compliant [33]. This may suggest that older mothers may feel that they have more experience with raising children than younger mothers. Therefore, they may perceive themselves as experts in their child’s health needs, whereby they can teach and train their child to achieve developmental tasks prior to the external intervention [34]. Children born in a complex delivery process may already be under close medical monitoring. This may be the reason that fewer parents comply with suggestions for ST at the MCHC.

Studies found disparities between noncitizen children and US citizen children under age six, in adherence to well-child visit recommendations [1,8]. The current study found that foreign children are more likely to comply with ST at the MCHC. This may indicate that the foreign community in Israel perceives the MCHC as providing important preventive healthcare services, including the vaccination program [21]. It may be assumed that the foreign community, which has poor health insurance, perceives the MCHC as the first and only high-quality community medical advice that they can receive free-of-charge.

Analyzing differences between children whose parents acted differently on physician/nurse referrals indicated no significant differences aside from low responses among parents of children with a higher number of children in the family and high responses among parents of children for whom the finding of an abnormality was initially uncovered by a physician in the MCHC. A large family with a higher number of children was found to be a barrier to meeting the child’s health needs, including preventive healthcare needs [8,11,35]. This finding suggests that the child’s health needs are deferred due to family difficulties. Notwithstanding this barrier, the initial diagnosis in the MCHC encourages parents to utilize the referral they are given. Fuzzell et al. (2018) [36] found that a recommendation of health needs and safety guidelines by the health provider predicts greater adherence to the recommendations by parents. When a physician/nurse’s suspicion of a health or developmental problem associated with the child continues some concern of the parents, this enhances the parent’s inclination to comply with the referral [34]. Sometimes the parents cannot see or recognize the symptoms of the abnormality (“non-presenting symptom”) [37] and when the MCHC physician initially highlights the problem, it may shock the parents and motivate them to act on the referral.

Menahem and Halase (2000) [37] argued that the presentation of a health problem to the parents should be based on a good doctor/nurse–patient relationship, good communication, and trust. This approach will help the parents cope with their anxiety by acting on the referral for their child’s wellbeing. Perry et al. (2017) [38] recommended four strategies for health intervention with the aim of improving neonatal and child health. One of these is to deepen and widen the recognition and referral of children with health and developmental problems for further evaluation and treatment. All of this emphasizes the need to increase the knowledge and practice of adhering to formal recommendations [2,26]; to market the importance of ST and of adhering to referrals [39] using good-quality open communication with the parents [7,37]; to use valid tools for ST with good physician/nurse skills and confidence [40,41]; to deepen the knowledge of physicians in the field of child development and community care options [26]; and most importantly, to recognize families with difficulties and help them deal with their challenges [37,38]. Insights regarding the current research findings may suggest that to mitigate parents’ nonadherence with screening tests and further referrals, good communication between the physicians/nurses and parents should be adopted, while at the same time encouraging parents’ high confidence and trust in the health staff at MCHCs. The parents should be convinced of the significance and consequences of the screening tests for their child’s health throughout their life [7]. Health policymakers need to mediate and convince parents of the importance of conducting monitoring, screening tests, and follow-up, particularly in infants and toddlers, in order to take advantage of the window of opportunity for good health throughout a child’s life and community health [29].

This study had several limitations. First, the study was conducted among children in a single city and may therefore not represent all the children in Israel. However, the city is the second largest in Israel and encompasses a variety of communities on different socioeconomic levels. Second, there were only a small number of referrals. We recommend extending the study to other cities in Israel. Finally, the data were extracted from the children’s medical files. There may be differences in the recording methods between the physicians or the nurses. Nevertheless, all MCHCs are managed by one professional body (the municipal public health department) and follow the procedures of the Ministry of Health concerning computerized medical records. These facts reduce the likelihood of record differences.

## 5. Conclusions

The current study indicates that parents of young children show high compliance with childhood ST, but it is higher among parents of two-month-old children than among those of nine-month-old children. There are several potential factors associated with not undergoing STs: a complex delivery process, older mothers, a large family, and being Israeli citizens. A lower number of children in the family and an initial finding in the MCHC are the only explanation for parents’ adherence to further referrals.

A practical implication of the current study should be the need to focus on reducing the rate of children who do not undergo STs, particularly older children. Healthcare policymakers should develop an explanatory campaign addressing the importance of conducting STs in early childhood, as well as the importance of early diagnosis and treatment for achieving a better prognosis for the child. A sophisticated social media campaign should be employed in order to reach specific parents, such as families with a high number of children or older parents. It is important to encourage open communication, trust, and confidence between the parents and the physicians and nurses, which makes it possible to deal with obstacles to carrying out the professional’s recommendations. All this together may promote higher parent compliance with ST and with further referrals and treatment. This is essential for ensuring the health and wellbeing of the child throughout his life cycle and a future healthy community.

The study was conducted prior to the COVID-19 pandemic. Further research should examine the impact of COVID-19 on adherence to screening tests in MCHCs.

## Figures and Tables

**Table 1:** Comparison between the 2016 and 2017 cohorts who underwent physician and nurse screening tests.

	2016 Cohort (n = 397)	2017 Cohort (n = 447)	χ^2^	*p* Value
Physician’s screening test	N (%)	N (%)		
Underwent physician’s screening test			9.98	0.002
Yes	289 (72.8)	366 (81.9)		
No	108 (27.2)	81 (18.1)		
Nurse’s screening test	N (%)	N (%)		
Underwent nurse’s screening test			33.02	0.001
Yes	348 (87.7)	437 (97.8)		
No	49 (12.3)	10 (2.2)		

**Table 2:** Screening test outcomes for the 2016–2017 cohort, physicians and nurses.

	Physician’s Screening Test N (%)	Nurse’s Screening Test N (%)	χ^2^	*p*<
Performed			134.3	0.0001
Yes	655 (77.6)	785 (93.0)		
No	189 (22.4)	59 (7.0)		
Outcome			3.29	0.07
No finding	393 (60.0)	692 (88.4)		
Suspicious finding	262 (40.0)	91 (11.6)		
Types of findings:				
General condition	46 (17.4)			
Skin system	62 (23.5)			
Head, face, neck	63 (23.9)			
Chest	14 (5.3)			
Cardiovascular system	15 (5.6)			
Stomach	19 (7.2)			
Genital system	18 (6.8)			
Bones	21 (8.0)			
Neurological system	6 (2.2)			
Growth problem	-	73 (80.2)		
Developmental problem	-	18 (19.8)		
Initial finding				
Yes	137 (52.3)	-		
No	84 (32.1)			
Unknown	41 (15.6)			
Referral ^a^			54.63	0.0001
Yes	94 (35.9)	79 (86.8)		
No	168 (64.1)	12 (13.2)		
Parents acted on the referral			14.43	0.0001
Yes	61 (64.9)	70 (88.6)		
No	33 (35.1)	9 (11.4)		
Referral Outcome ^b^			0.00	0.98
No finding	29 (47.5)	34 (48.6)		
Pathological finding	32 (52.5)	36 (51.4)		

^a^ Referral of children who had suspicious findings (n = 264 for physicians and n = 91 for nurses). ^b^ Referral outcome for children whose parents acted on the referral (n = 61 for physicians and n = 70 for nurses).

**Table 3:** Differences between children who did or did not undergo a physician’s or nurse’s screening test, N = 844.

	Physician’s Screening Test		Nurse’s Screening Test	
	Underwent N (%)	Did Not Undergo N (%)	Underwent N (%)	Did Not Undergo N (%)
Gender ^a^				
Female	316 (75.2)	104 (24.8)	384 (91.4)	36 (8.6)
Male	339 (80.0)	85 (20.0)	401 (94.6)	23 (5.4)
Number of children in pregnancy ^a^				
1	626 (78.3)	28 (65.1)	743 (92.9)	57 (7.1)
2 or more	174 (21.8)	15 (34.9) *	41 (95.3)	2 (4.7)
Delivery process ^a^				
Vaginal	432 (75.3)	142 (24.7)	531 (92.5)	43 (7.5)
Caesarian/complex	221 (82.5)	47 (17.5) *	252 (94.0)	16 (6.0)
Infant medical problem at delivery ^a^				
No finding	455 (77.9)	129 (22.1)	537 (92.0)	47 (8.0)
Pathological findings	196 (76.9)	59 (23.1)	243 (95.3)	12 (4.7)
Mother’s age, M(SD) ^b^	33.4 (5.53)	35.1 (5.32) ***	33.6 (5.50)	36.2 (5.28) ***
Number of children in the family, M (SD) ^b^	1.9 (1.25)	2.3 (1.29) ***	1.9 (1.28)	2.2 (1.19)
Marital status ^a^				
Married	570 (77.0)	170 (23.0)	685 (92.6)	55 (7.4)
Not married	83 (82.2)	18 (17.8)	97 (96.0)	4 (4.0)
Citizenship ^a^				
Israeli citizen	529 (74.8)	178 (25.2)	649 (91.8)	58 (8.2)
Foreign worker/asylum seeker	124 (91.9)	11 (8.1) ***	134 (99.3)	1 (7.0) **
Education level ^a^				
Not academic	238 (82.0)	62 (18.0)	327 (94.8)	18 (5.2)
Academic	361 (74.1)	126 (25.9) **	447 (91.8)	40 (8.2)
Religion ^a^				
Jewish	480 (75.2)	158 (24.8)	585 (91.7)	53 (8.3)
Arab	175 (85.0)	31 (15.0) **	200 (97.1)	6 (2.9) **

^a^ Chi-square, ^b^ Student’s *t*-test. * *p* < 0.05; ** *p* < 0.01; *** *p* < 0.001.

**Table 4:** Multiple logistic regression for the association between the research variables and not undergoing a physician’s or nurse’s screening test at a MCHC ^a^.

	Predictor: Physician’s Screening Test		Predictor: Nurse’s Screening Test	
	Adjusted OR	95% CI	Adjusted OR	95% CI
Number of children in pregnancy			-	-
One child—ref	1			
Two or more	0.05	0.25–1.02		
Mother’s age	0.96	0.93–0.99 *	0.93	0.88–0.98 **
Delivery process	1		-	-
Vaginal—ref				
Caesarian/complex	1.70	1.14–2.54 **		
Number of children in the family	0.84	0.72–0.97 *	-	-
Citizenship				
Israeli citizen—ref	1		1	
Foreign worker/asylum seeker	3.95	1.73–9.04 ***	10.33	1.18–0.98 *
Education level			-	-
Not academic—ref	1			
Academic	0.84	0.55–1.28		
Religion				
Jewish—ref	1		1	
Arab	1.51	0.83–2.75	1.16	0.43–3.13

^a^ The table shows two logistic regressions, one for a physician’s screening test and the other for a nurse’s screening test. The dependent variable for both logistic regression equations was “did/did not undergo the screening test” (1/0, respectively). The independent variables were variables that were significant in previous statistical tests (shown in Table 3), that were included in the logistic regression for both a physician’s and nurse’s screening test. CI is the confidence interval for the odds ratio (OR). * *p* < 0.05; ** *p* < 0.01; *** *p* < 0.001.

**Table 5:** Parents’ acting on physician’s or nurse’s referral ^a^.

	Physician’s Referral (n = 94)		Nurse’s Referral (n = 79)	
	Took Action N (%)	Did Not Take Action N (%)	Took Action N (%)	Did Not Take Action N (%)
Gender				
Female	24 (58.8)	17 (41.5)	36 (87.8)	5 (12.2)
Male	37 (69.8)	16 (30.2)	34 (89.5)	4 (10.5)
Delivery process				
Vaginal	31 (62.0)	19 (38.0)	45 (86.5)	7 (13.5)
Caesarian/complex	30 (68.2)	14 (31.8)	25 (96.2)	1 (3.8)
Infant medical problem at delivery				
No finding	39 (62.9)	23 (37.1)	46 (86.6)	7 (13.2)
Pathological findings	22 (71.0)	9 (29.0)	24 (92.3)	2 (7.7)
Mother’s age, M (SD)	33.1 (6.62)	33.7 (5.32)	33.9 (4.95)	33.3 (4.58)
Number of children in the family, M (SD)	1.6 (0.73)	2.06 (0.99) *	1.8 (0.86)	1.6 (0.52)
Origin				
Israeli citizen	42 (60.9)	27 (39.1)	54 (88.5)	7 (11.5)
Foreign worker/asylum seeker	19 (76.0)	6 (24.0)	16 (88.9)	2 (11.1)
Education level				
Not academic	33 (63.5)	19 (36.5)	35 (94.6)	2 (5.4)
Academic	27 (67.5)	13 (32.5)	35 (83.3)	7 (16.7)
Religion				
Jewish	37 (63.8)	21 (36.2)	51 (87.9)	7 (12.1)
Arab	24 (66.7)	12 (33.3)	19 (90.5)	2 (9.5)
Initial finding				
Yes	53 (70.7)	22 (29.3)		
No	8 (42.1)	11 (57.9) *	-	-

^a^ The table shows differences between two groups: parents who took action following the physician and nurse’s referral and parents who did not take action. * *p* < 0.01.

## Data Availability

The data presented in this study are available on request from the corresponding author. The data are not publicly available due to private personal information of the children.

## Appendix A

**Table A1:** Sample characteristics (n = 844).

Variable	N (%)	M (SD)	Median
Child’s information			
Gender			
Female	420 (49.8)		
Male	424 (50.2)		
Number of children in pregnancy			
1	800 (94.9)		
2	42 (5.0)		
3	1 (0.1)		
Delivery process *			
Vaginal	574 (68.0)		
Caesarian/complex	268 (31.8)		
Infant medical problems at delivery			
No finding	584 (69.6)		
Abnormal findings **	255 (30.4)		
Birth age (weeks)		39.2 (2.03)	39.7
Mother’s information			
Age		33.8 (5.52)	34.0
Number of children in the family		1.96 (1.27)	2.0
Marital status			
Married	740 (88.0)		
Not married	101 (12.0)		
Citizenship			
Israeli citizen	707 (84.0)		
Foreign worker/asylum seeker	135 (16.0)		
Education level			
Not academic	345 (41.5)		
Academic	487 (58.5)		
Religion			
Jewish	638 (75.6)		
Muslim	61 (7.2)		
Christian	139 (16.5)		
Other	6 (0.7)		

* Numbers that do not add up to 100%, meaning missing data. ** Abnormal findings, for example, premature, G6PD, IUGR, VSD, PFO, torticollis, tongue-tied, coffee olla spot, systolic murmur, undescended testicles, respiratory distress, hemangiomas, skin tag, hydrocephalus, meconium amniotic fluid.
